# Depression and weight loss trajectories during an integrated behavioral intervention: Within-treatment analysis of the RAINBOW trial

**DOI:** 10.1371/journal.pone.0328715

**Published:** 2025-12-19

**Authors:** Bailey W. Osweiler, Daphne Lew, Nan Lv, Jun Ma, Thomas G. Kannampallil

**Affiliations:** 1 Division of Computational and Data Sciences, McKelvey School of Engineering, Washington University in St. Louis, St. Louis, Missouri, United States of America; 2 Institute for Informatics, Data Science, & Biostatistics (I2DB), School of Medicine, Washington University in St. Louis, St. Louis, Missouri, United States of America; 3 Center for Health Behavior Research, School of Public Health, University of Illinois Chicago, Chicago, Illinois, United States of America; Iranshahr University of Medical Sciences, IRAN, ISLAMIC REPUBLIC OF

## Abstract

**Introduction:**

Depression and obesity are two of the most common chronic conditions among US adults. The Research Aimed at Improving Both Mood and Weight (RAINBOW) study was the first randomized clinical trial to demonstrate the effectiveness of an integrated behavioral treatment for depression and obesity. We aimed to characterize the trajectories of both depression symptoms and weight loss during the RAINBOW intervention.

**Methods:**

Participants (N = 201) randomly assigned to the RAINBOW treatment arm who provided serial data on depression symptoms (via the 9-item Patient Health Questionnaire [PHQ9]) and self-measured weight were included. Group-Based Multi-Trajectory Modeling is a statistical technique that identifies participants who follow similar trajectories of multiple outcomes over time. This approach was used to characterize latent groups of both PHQ9 and weight loss percentage during treatment. Demographic differences between groups were investigated using Chi-Square, Fisher’s exact tests, or ANOVA tests. Group membership was investigated for relationships with 12-month change in independently measured depression (via the 20-item Symptom Checklist, or SCL20) and weight (measured by trained research staff) using linear regression models.

**Results:**

Participants were classified into 3 groups based on their depression symptom and weight trajectories: Moderate Depression Improvements/Minimal Weight Loss (N = 108, 53.7%), Substantial Depression Improvements/Moderate Weight Loss (N = 58, 28.9%), and Substantial Depression Improvements/Substantial Weight Loss (N = 35; 17.4%). Older participants were more likely to be in the Substantial/Substantial group. Participants in the Substantial/Moderate and Substantial/Substantial groups had larger 12-month depression symptom score changes and 12-month weight loss compared to the Moderate/Minimal group.

**Conclusion:**

We identified three joint trajectories of depression symptom scores and obesity. Members in the three trajectories saw varying degrees of symptom reduction from baseline to 12 months. Results suggest adaptive treatment trials may be needed for participants in groups with lower anticipated depression symptom scores and/or weight reductions.

## Introduction

Depression and obesity are two of the most common chronic conditions among US adults [1]. Recent studies have estimated that over 40% of US adults are obese [2], and 8% of adults (~21 million) had at least one major depressive episode in the past year [3]. Furthermore, a 2016 systematic review found that people with obesity were 32% more likely to have depression compared to healthy patients, and this comorbidity is especially pronounced among women [4]. Efficacious treatments exist for depression and obesity individually [5,6], but an integrated, efficient, and cost-effective approach is needed to treat these two conditions when they co-occur [7].

Several studies have used trajectory analyses to separately assess depression and obesity symptoms and outcomes, which helps assess the heterogeneity in treatment response and identify groups of patients with persistent symptoms [8–15]. A recent scoping review found that trajectory modeling has revealed lifetime obesity trends in many studies [10]. The method is also used to map symptom trajectories during treatment, such as investigating heterogeneity in a community-based non-diet weight intervention [9], and weight outcomes during randomized clinical trials [8,15]. As described in Song et al. [14] regarding life course obesity trajectories in epidemiology, identifying groups can set the foundation for translational research on consequences of these treatment trajectories. Additionally, studies have shown distinct depression symptom trajectories among patient populations including primary care [11], older adults [12], patients treated for co-occurring anxiety and depression [16], and patients with Lupus [13].

The Research Aimed at Improving Both Mood and Weight (RAINBOW) study was the first randomized clinical trial to demonstrate the effectiveness of an integrated behavioral treatment for depression and obesity [7]. Longitudinal clustering methods showed that RAINBOW participants with poor treatment engagement can be identified early and have worse depression and obesity outcomes than participants with high treatment engagement [17]. However, this analysis investigated the two outcomes separately, making it impossible to differentiate covarying patterns across the outcomes; for example, participants may follow similar trajectories on one outcome (e.g., depression) while differing on the other (e.g., weight loss).

The RAINBOW trial provides a unique dataset where trajectory analysis can be used to contextualize an integrated behavioral treatment by identifying participant subgroups with varying degrees of treatment success for both conditions. In this post-hoc study, we hypothesized that participants would exhibit distinct trajectories of depression symptoms and weight loss over the course of 12 months of treatment.

## Methods

### Original study: participants

Data for this secondary analysis was from the RAINBOW clinical trial, which took place in Northern California between September 2014 and January 2017 [7]. The original study included adults with elevated depressive symptoms, defined as a Patient Health Questionnaire (PHQ9) score ≥ 10, and co-occurring obesity, defined as Body Mass Index (BMI) ≥ 30, or ≥ 27 for Asian participants. The original study obtained written informed consent from participants and was approved by the Institutional Review Board at Sutter Health, North California. The original trial protocol was published [18]. Data was anonymized before use in this secondary analysis, which began January 26, 2024.

### Original study: intervention

Participants were randomly assigned to the treatment group (n = 204) and received an integrated intervention including multiple sessions of Problem-Solving Therapy (PST) for depression and Group Lifestyle Balance (GLB) for weight loss for one year. PST for depression consisted of 15 one-on-one live coaching sessions with a trained professional, through an adapted version of the Program to Encourage Active, Rewarding Lives for Seniors [19]. The first 9 PST sessions were delivered in a 6-month intensive treatment phase, which were face-to-face 60-minute sessions at first weekly (4), then biweekly (2), then monthly (3). The remaining 6 sessions in the maintenance phase were monthly 15- to 30-minute telephone calls with brief mood and weight sessions. The GLB intervention was an adapted version of the Diabetes Prevention Program and included self-study videos at home [20]. The intensive treatment phase included 11 home-viewed GLB videos. No videos were watched during the maintenance phase.

More information on the integrated intervention is available in the original study [7] and accompanying articles [19,20]. The RAINBOW trial demonstrated significantly greater improvements in both depressive symptoms and weight loss in the treatment versus the usual care group at 12-month follow up [7].

### Current study: data

Anonymized data from the RAINBOW treatment group participants were used for this post-hoc analysis. Three participants with fewer than two depression or weight measurements were removed. A sensitivity analysis was conducted removing individuals with two measurements or less, to determine whether the exclusion of subjects with fewer than three measurements meaningfully impacted the results. Data included repeated measures of depression symptoms and weight by self-report for trajectory classification, the baseline and 12-month depression symptoms and weight measures by trained research staff, and covariates as outlined below. The final analytic dataset for the trajectory modeling included one row for each unique participant-week with at least one observation for either depression or weight. For the 15 PST session weeks, depression symptoms and weight measurements were linked to the PST session number and anchored to the week they were expected to be performed. All other self-measured weight observations recorded their week number.

### Current study: measures

#### Primary outcomes.

The primary outcomes were depression symptom scores and weight changes between baseline and 12-month follow-up per participant. Final depression symptomology was measured using the Symptom Checklist Depression Scale (SCL20), a 20-question depression severity questionnaire that has been proven to be valid and reliable [21]. Weight was measured by trained study staff following a standardized protocol [7].

#### Secondary outcomes.

Both primary outcomes were classified into binary categorical variables indicating whether the change was clinically significant. Clinically significant depression symptomology improvement was considered a 50% reduction from baseline to 12 months (i.e., depression response) or SCL20 < 0.5 at 12 months (i.e., depression remission) [7,22], and clinically significant weight loss was considered a 5% reduction from baseline to 12 months [23,24].

#### Depression symptoms over time.

The longitudinal data included the 9-item PHQ9 measurements recorded at screening and at the beginning of each of the 15 PST sessions, to capture repeated depression symptom scores separately from the final depression score assessment (SCL20). The PHQ9 is a short-form, self-administered assessment for depression severity which asks the patient 9 questions about their DSM-V depression criteria, with each question having a score range between “0” (not at all) and “3” (every day) [25–28]. Each composite PHQ9 score is an integer between 0 and 27.

#### Weight over time.

The longitudinal data included participants’ weekly self-monitored weight, measured in kilograms, for a maximum of 54 consecutive weeks. Each weekly weight measurement was transformed as a continuous percent change from baseline, with negative values indicating more “success” or greater weight loss, using the following formula:


$$percent\;weight\;change\;=\frac{\left(weekly\;weight\;-baseline\;weight\right)}{baseline\;weight}\;*\text{1}\text{0}\text{0}$$

#### Demographics.

Sex, race (Non-Hispanic White, Hispanic, Asian, Black, Other), age, educational attainment (<high school, some college, undergraduate degree, or graduate degree), income range ($0-$34999, $35000-$74999, $75000-$99999, $100000-$149000, and>$150000), and marital status (single vs married or living with another person) were collected at baseline.

#### Co-occurring conditions.

Co-occurring mental illness was measured for post-traumatic stress disorder (PTSD) via the PTSD Checklist [29] and generalized anxiety disorder via the 7-item Generalized Anxiety Disorder scale (GAD7) [30]. Other health information included the Sheehan Disability Score [31], both systolic blood pressure (SBP) and diastolic blood pressure (DBP), 24-hour caloric intake recall, 7-day physical activity recall, obesity-related problems score, current binge eating disorder, and lifetime or current panic disorder. Whether the patient was taking any antidepressant medication was also recorded. All co-occurring conditions and medications were assessed at baseline.

### Current study: statistical analysis

Descriptive statistics of mean, standard deviation (SD), frequencies, and proportions were calculated for the entire sample and all sub-groups. To compare differences in categorical demographic variables and comorbidities (e.g., sex, race, binge eating disorder), we performed Chi-Square or Fisher’s Exact tests (as appropriate based on expected cell frequencies). To compare differences in means of continuous, normally distributed demographic and clinical variables (e.g., age, blood pressure) across groups, we performed ANOVA tests. For all statistical tests, the identified trajectory group was treated as the independent variable of interest.

Group-Based Multi-Trajectory Modeling (GBMTM) was used to characterize latent groups among the treatment group participants. Group-Based Trajectory Modeling [32] is a maximum likelihood estimation technique that groups participants that follow similar trajectories over time. The multi-trajectory extension of this method allows for the identification of groups that follow similar trajectories for multiple outcomes (in our case, depression symptoms and weight) [32]. This approach is robust to missingness in one or both repeated measure variables, which is important in this analysis due to high missingness in the self-measured weight data. GBMTM can also account for inconsistent follow-up times. Both depression symptom scores and weight change were modeled as normally distributed, continuous variables. Though the PHQ-9 used to measure depression symptom scores is a discrete integer, our visual inspection of the distribution of the data supports the decision to model it as continuous for the purposes of these analyses. Further, the overall scale score has been commonly used as a continuous variable [25] and simulation studies have shown that when the number of discrete values is large (11 or more), the underlying distribution will approach normality [33,34]. The analysis was performed using the R multlcmm package [35] and R version 3.4.1 [36].

GBMTM models using one through five groups were assessed. Model selection was conducted using the Akaike Information Criterion (AIC) fit statistic and conceptual interpretation of the groups. In particular, the elbow test was applied to the AIC by creating a plot of the fit statistic values versus the number of trajectory groups in the model. Then, the “elbow” of the plot was identified, where increases in trajectory groups no longer corresponded to dramatic decreases in AIC. Participants were classified into the multi-trajectory group with the highest probability of group membership, and sensitivity analysis was performed by creating a subset of the data that only included individuals with group membership probability of at least 0.5 or 0.7. We investigated patterns of missingness for repeated measures and outcomes as additional sensitivity analysis.

To estimate the relationship between trajectory group membership and 12-month outcomes, two linear regression models were fitted to separately predict change in SCL20 and percent change in weight from baseline to 12 months. Models included one observation per participant, with the primary independent variable of interest being trajectory group membership, and controlled for baseline SCL20 or weight (respectively) and any demographic characteristics that were found to significantly differ between groups. Effect estimates from these linear regression models were calculated as beta coefficients with 95% confidence intervals (CIs).

To examine differences in clinically significant changes in outcomes across trajectory groups, two separate logistic regression models were fit. Models controlled for baseline SCL20 or weight (respectively) and the same demographic characteristics as the previous models. The binary outcomes of the two models were clinically significant change in SCL20 and weight from baseline to 12 months (as described in the “Secondary Outcomes” section above). Effect estimates from these logistic regression models were calculated as odds ratios (ORs) with 95% CIs.

## Results

### Descriptive characteristics

Of the 204 participants in the treatment group, three participants were removed due to only having one measurement for PHQ9 and weight. This resulted in a final analytic sample of 201 participants. The mean age was 50.8 (SD = 12.2), 70.6% (n = 142) were female, and 72.6% (n = 146) of the sample was non-Hispanic White. The mean SCL20 score at baseline was 1.45 (SD = 0.53) and the mean weight was 104 kilograms (SD = 22.4). See Table 1 for further descriptive characteristics of participants.

**Table 1:** Summary statistics of demographics and comorbidities across 3 identified groups.

	Overall (N = 201)	Moderate/ Minimal (N = 108)	Substantial/ Moderate (N = 58)	Substantial/ Substantial (N = 35)	*p*
**Baseline Weight**					
Mean (SD)	104 (22.4)	104 (24.3)	106 (21.0)	97.1 (16.9)	0.222 (c)
[Min, Max]	[61.1, 203]	[61.1, 203]	[64.3, 182]	[75.3, 151]	
**Baseline SCL20**					
Mean (SD)	1.45 (0.532)	1.43 (0.515)	1.52 (0.574)	1.44 (0.517)	0.665
[Min, Max]	[0.300, 2.65]	[0.350, 2.60]	[0.300, 2.65]	[0.700, 2.25]	
**Sex**					
Women	142 (70.6%)	81 (75.0%)	38 (65.5%)	23 (65.7%)	0.344
Men	59 (29.4%)	27 (25.0%)	20 (34.5%)	12 (34.3%)	
**Race**					
Asian	18 (9.0%)	11 (10.2%)	3 (5.2%)	4 (11.4%)	0.574
Hispanic	26 (12.9%)	17 (15.7%)	6 (10.3%)	3 (8.6%)	
Non-Hispanic White	146 (72.6%)	76 (70.4%)	44 (75.9%)	26 (74.3%)	
Other	8 (4.0%)	2 (1.9%)	4 (6.9%)	2 (5.7%)	
Black	3 (1.5%)	2 (1.9%)	1 (1.7%)	0 (0%)	
**Age**					
Mean (SD)	50.8 (12.2)	49.2 (12.6)	51.6 (11.2)	54.3 (11.9)	0.024 * (b)
[Min, Max]	[20.0, 76.0]	[20.0, 74.5]	[27.7, 76.0]	[22.9, 69.6]	
**Education**					
<High school or GED	10 (5.0%)	6 (5.6%)	1 (1.7%)	3 (8.6%)	0.177
Some college	51 (25.4%)	33 (30.6%)	14 (24.1%)	4 (11.4%)	
Undergraduate degree	77 (38.3%)	39 (36.1%)	25 (43.1%)	13 (37.1%)	
Graduate work or degree	63 (31.3%)	30 (27.8%)	18 (31.0%)	15 (42.9%)	
**Income**					
$0 - $35,000	10 (5.0%)	6 (5.6%)	1 (1.7%)	3 (8.6%)	0.5542
$35,000 - $74,999	36 (17.9%)	23 (21.3%)	8 (13.8%)	5 (14.3%)	
$75,000 – $99,999	20 (10.0%)	12 (11.1%)	4 (6.9%)	4 (11.4%)	
$100,000 – $149,999	34 (16.9%)	17 (15.7%)	11 (19.0%)	6 (17.1%)	
> $150,000	74 (36.8%)	40 (37.0%)	22 (37.9%)	12 (34.3%)	
Missing	27 (13.4%)	10 (9.3%)	12 (20.7%)	5 (14.3%)	
**Marital Status**					
Married or living with another person	120 (59.7%)	62 (57.4%)	39 (67.2%)	19 (54.3%)	0.4046
Single	80 (39.8%)	46 (42.6%)	19 (32.8%)	15 (42.9%)	
Missing	1 (0.5%)	0 (0%)	0 (0%)	1 (2.9%)	
**SBP**					
Mean (SD)	119 (12.2)	118 (12.3)	122 (10.7)	120 (13.8)	0.251 (a)
[Min, Max]	[90.0, 160]	[90.0, 159]	[103, 147]	[90.0, 160]	
**DBP**					
Mean (SD)	79.0 (8.85)	78.3 (9.16)	81.4 (7.87)	77.3 (8.85)	0.851 (a) (c)
[Min, Max]	[58.7, 104]	[60.7, 104]	[65.3, 99.3]	[58.7, 96.0]	
**Sheehan Disability**					
Mean (SD)	11.8 (6.90)	11.3 (6.45)	13.0 (7.53)	11.6 (7.15)	0.500
[Min, Max]	[0, 30.0]	[0, 30.0]	[0, 27.0]	[0, 27.0]	
Missing	3	2	1	0	
**Calories**					
Mean (SD)	1810 (797)	1790 (712)	1940 (985)	1650 (679)	0.687
[Min, Max]	[273, 5150]	[273, 4250]	[621, 5150]	[618, 3170]	
Missing	2	1	1	0	
**Physical Activity**					
Mean (SD)	33.4 (2.34)	33.4 (2.30)	33.5 (2.81)	33.1 (1.50)	0.546
[Min, Max]	[28.8, 48.0]	[30.5, 43.8]	[28.8, 48.0]	[30.5, 36.7]	
**Obesity Problems Score**					
Mean (SD)	67.8 (24.3)	67.0 (25.1)	69.8 (20.9)	67.2 (27.2)	0.792
[Min, Max]	[0, 100]	[0, 100]	[16.7, 100]	[8.33, 100]	
**Binge Eating Disorder**					
Yes	76 (37.8%)	38 (35.2%)	25 (43.1%)	13 (37.1%)	0.602
No	125 (62.2%)	70 (64.8%)	33 (56.9%)	22 (62.9%)	
**Panic Disorder**					
None	165 (82.1%)	88 (81.5%)	45 (77.6%)	32 (91.4%)	0.452
Lifetime	20 (10.0%)	11 (10.2%)	8 (13.8%)	1 (2.9%)	
Limited symptom attacks lifetime	1 (0.5%)	1 (0.9%)	0 (0%)	0 (0%)	
Current	11 (5.5%)	5 (4.6%)	5 (8.6%)	1 (2.9%)	
Missing	4	3	0	1	
**Any antidepressant medications**					
Yes	24 (11.9%)	9 (8.3%)	9 (15.5%)	6 (17.1%)	0.211
No	177 (88.1%)	99 (91.7%)	49 (84.5%)	29 (82.9%)	
**PTSD Score**					
Mean (SD)	38.3 (12.7)	37.8 (12.8)	40.0 (13.6)	36.8 (11.0)	0.996
[Min, Max]	[17.0, 78.0]	[21.0, 75.0]	[17.0, 78.0]	[19.0, 59.0]	
Missing	2	1	0	1	
**GAD7 Score**					
Mean (SD)	8.20 (5.01)	8.12 (4.92)	8.40 (4.83)	8.11 (5.65)	0.921
[Min, Max]	[0, 21.0]	[0, 21.0]	[0, 19.0]	[0, 21.0]	
Missing	1	1	0	0	
**Alcohol Use**					
Yes	108 (53.7%)	60 (55.6%)	29 (50.0%)	19 (54.3%)	0.905
No	70 (34.8%)	38 (35.2%)	19 (32.8%)	13 (37.1%)	
Missing	23	10	10	3	
**Tobacco Use**					
Yes	8 (4.0%)	4 (3.7%)	4 (6.9%)	0 (0%)	0.643
Quit	49 (24.4%)	27 (25.0%)	14 (24.1%)	8 (22.9%)	
Never	139 (69.2%)	77 (71.3%)	37 (63.8%)	25 (71.4%)	
Missing	5	0	3	2	

*difference in three group comparison

(a) bivariate difference between Moderate/Minimal group and Substantial/Substantial group

(b) bivariate difference between Moderate/Minimal group and Substantial/Moderate group

(c) bivariate difference between Substantial/Moderate group and Substantial/Substantial group All comparisons use significance level ofα

### Multi-trajectory modeling

Based on the model fit statistics, two, three or four group models were examined as candidates for the final model because they had the lowest AIC values (S1 Fig, S1 Table). From these options, the three-group model was selected, as the two-group model missed granularity between groups and the four-group model created a small (n = 14) group that was a subset of one from the three-group model. S2 Table in the appendix shows the average assignment probability for each group. Sensitivity analyses excluding individuals with only two measurements (n = 7) yielded no meaningful differences in the identified trajectories.

**Fig 1:**
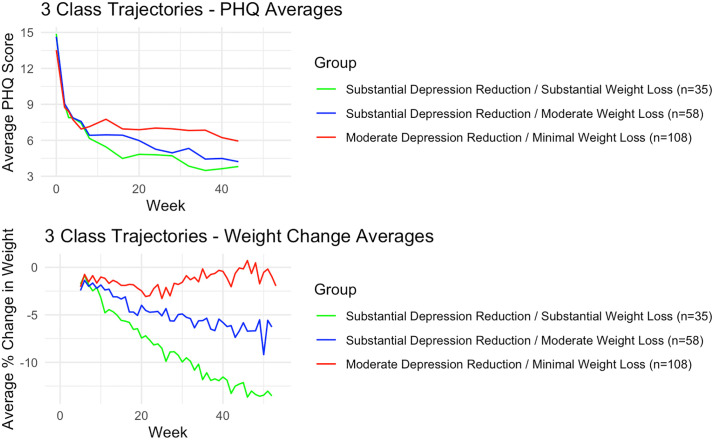
Average trajectories of 3-group GBMTM. PHQ and weight loss trajectories.

From this model, three groups emerged (Fig 1, S2 Fig, S3 Fig). The first and largest group (n = 108) showed moderate depression symptom score reductions but almost no weight loss over the study period. The second group (n = 58) experienced substantial depression symptom score reductions and a moderate, steady weight loss. The third group (n = 35) experienced substantial depression symptom reductions – comparable to group 2 – and a substantial, steady weight loss. Based on these characteristics, the three groups were named as follows: Group 1 as “Moderate/Minimal” group, group 2 as “Substantial/Moderate” group, and group 3 as “Substantial/Substantial” group. For each group name, the first descriptor represents the change in depression symptom scores and the second descriptor represents the weight loss.

### Demographics across trajectory groups

The sociodemographic characteristics and comorbidities of the three groups were similar for all considered characteristics except for age. There was a statistically significant association between age and group membership (p = 0.024), with the Substantial/Substantial group having the oldest participants (mean: 54.3, SD: 12.9) and the Moderate/Minimal group having the youngest (mean: 49.2, SD: 12.6). Characteristics of all trajectory groups can be found in Table 1.

### Relationship between trajectory groups and primary outcomes

All three groups saw an average reduction in depression symptomology from baseline to 12 months but differed in the magnitude of the change: the Moderate/Minimal group had a 0.08 reduction (SD = 0.81), the Substantial/Moderate group a 0.46 reduction (SD = 0.60), and the Substantial/Substantial group a 0.55 reduction (SD = 0.63; Table 2). In the multivariable model, trajectory group membership was found to be significantly related to change in depression symptomology, after controlling for baseline symptoms and age. Compared to the Moderate/Minimal group, the Substantial/Moderate group had an average additional 0.31 (95% CI: −0.54, −0.08) reduction in SCL20 at 12 months, and the Substantial/Substantial group had an average additional 0.47 (95% CI: −0.75, −0.19) reduction in SCL20 at 12 months.

**Table 2:** Regression results for 12-month depression and weight changes.

	Linear Regression – Change in Outcome			Logistic Regression – Clinically Significant (CS) Change in Outcome		
SCL20: observations with both baseline and 12-month SCL = 169						
*Predictors*	*Unadjusted SCL change (SD)*	*Adjusted Estimates (95% CI)*	*p*	*Proportion achieving CS change in SCL*	*Odds Ratios (95% CI)*	*p*
Baseline SCL20	–	−0.53 (−0.73, −0.33)	**<0.001**	–	1.12 (0.58, 2.19)	0.733
Age	–	−0.01 (−0.01, 0.00)	0.205	–	1.02 (0.99, 1.05)	0.159
Moderate/ Minimal (n = 85)	−0.08 (0.81)	Ref	Ref	0.22	Ref	Ref
Substantial/ Moderate (n = 53)	−0.46 (0.60)	−0.31 (−0.54, −0.08)	**0.010**	0.32	1.55 (0.71, 3.40)	0.268
Substantial/ Substantial (n = 31)	−0.55 (0.63)	−0.47 (−0.75, −0.19)	**0.001**	0.48	2.97 (1.23, 7.25)	**0.016**
Weight: observations with both baseline and 12-month Weight = 180						
*Predictors*	*Unadjusted Weight change* *(SD)*	*Adjusted Estimates* *(95% CI)*	*p*	*Proportion achieving CS change weight*	*Odds Ratios* *(95% CI)*	*p*
Baseline Weight (kg)	–	−0.03 (−0.06, 0.00)	0.070	–	1.00 (0.98–1.02)	0.999
Age	–	−0.01 (−0.07, 0.05)	0.634	–	1.01 (0.98–1.05)	0.491
Moderate/ Minimal (n = 95)	1.23 (5.00)	Ref	Ref	0.06	Ref	Ref
Substantial/ Moderate (n = 54)	−3.53 (4.40)	−4.64 (−6.25, −3.03)	**<0.001**	0.33	7.22 (2.77–21.37)	**<0.001**
Substantial/ Substantial (n = 31)	−9.55 (4.70)	−10.93 (−12.92, −8.95)	**<0.001**	**0.87**	94.56 (27.30–419.55)	**<0.001**

**BMI: observations with both baseline and 12-month BMI = 180**

With regards to weight outcomes, only two of the three groups experienced an unadjusted weight reduction from baseline to 12 months; the Moderate/Minimal Group had an average 1.23-kilogram weight gain (SD = 5.00), the Substantial/Moderate Group had an average 3.53-kilogram weight loss (SD = 4.40), and the Substantial/Substantial group had an average 9.55-kilogram weight loss (SD = 4.70; Table 2). In the multivariable model, the Substantial/Moderate group had an average additional 4.64-kilogram decrease (CI: −6.25, −3.03) and the Substantial/Substantial group had an average additional 10.93-kilogram decrease (CI: −12.92, −8.95) compared to the Moderate/Minimal group.

### Relationship between trajectory groups and clinically significant change

The proportion of participants achieving clinically significant change in depression symptoms (response or remission) ranged from 22% for the Moderate/Minimal group to 48% for the Substantial/Substantial group (Table 2). After controlling for covariates, only the Substantial/Substantial group, compared to the Moderate/Minimal group, was significantly associated with clinically significant change: the Substantial/Substantial group was 197% more likely (OR=2.97, CI: 1.23, 7.25) than the Moderate/Minimal group to achieve clinically significant SCL20 changes. The Substantial/Moderate group was more likely, but the results were not statistically significant (OR=1.55, CI: 0.71, 3.40).

For weight, only 6% of participants in the Moderate/Minimal group achieved clinically significant changes, compared to 33% in the Substantial/Moderate group and 87% in the Substantial/Substantial group (Table 2). After controlling for covariates, both the Substantial/Moderate group (OR=7.22, CI: 2.77, 21.37) and the Substantial/Substantial group (OR=94.56, CI: 27.30, 419.55) were significantly more likely to experience clinically significant weight loss at 12 months.

### Sensitivity analyses

Sensitivity analyses that included only individuals with high probability of trajectory group membership (> 50%, > 70%) were conducted. Results of sensitivity analyses were consistent with those described above: group membership was associated with 12-month change in SCL20 and weight, the Substantial/Moderate group was more likely to achieve clinically significant weight loss, and the Substantial/Substantial group was more likely to achieve both clinically significant weight loss and clinically significant SCL20 reductions (S3 Table, S4 Table, S5 Table, S4 Fig, S5 Fig). Analysis of repeated measures missingness over time (S6 Fig, S6 Table), and demographics across participants with and without outcomes (S7 Table) are available in the supplement.

## Discussion

This study contributes to the understanding of efficacy of RAINBOW, a joint behavioral treatment for comorbid depression and obesity, by identifying common trajectories of the two outcomes over the course of treatment. The majority (53.7%) of participants in RAINBOW saw modest depression symptom score improvements with no appreciable weight loss (the “Moderate/Minimal” group). Two other groups were also identified, which had both depression symptom score improvements and weight loss, but to varying degrees (the “Substantial/Moderate” and “Substantial/Substantial” groups). These results are consistent with previous work on longitudinal trends of depression change [11,16], weight change [8,15], and associated demographics [37,38], and extend the findings to model both outcomes jointly.

Participants in the Substantial/Substantial group experienced the largest improvements in both depression symptoms and weight loss. They were most likely to experience depression symptom score improvements and clinically significant depression symptom response, weight loss and clinically significant weight loss. Although the Substantial/Moderate group was more likely to experience any weight loss, clinically significant weight loss, and any depression symptom improvements than the Moderate/Minimal group, the clinically significant depression symptom improvements were not statistically significant. This may be explained by this group’s high baseline weight and SCL20 scores, given that clinical significance is partially defined by the baseline score. Moreover, the Substantial/Moderate group had the highest rates of many other measures such as blood pressure (SBP, DBP), caloric intake, obesity problems score, Sheehan disability score, Binge eating disorder, panic disorder, and PTSD. These differences between groups were not statistically significant, but it is possible that the Substantial/Moderate group was more clinically complex given their minimally higher values across many of these co-occurring measures. Nonetheless, the Substantial/Moderate group still showed depression symptom improvements and weight loss, potentially indicating that the RAINBOW intervention can be successful for individuals with both severe and moderate baseline characteristics, but to varying degrees.

Notably, participants in the “Moderate/Minimal” group were younger than participants in the other two groups, indicating that older age is likely associated with greater depression improvements and weight loss during an integrated treatment. This is consistent with other studies investigating interventions for weight loss alone, which have found that older participants experience faster weight loss [37]. Another recent meta-analysis revealed that older age is consistently associated with improved study adherence and retention in two longitudinal digital health studies [38], indicating a potential reason why older age may be associated with better RAINBOW outcomes. Our analysis expands the understanding of RAINBOW efficacy and can help cater this intervention to those that will benefit the most.

Our study builds on existing research on both depression and obesity by identifying concurrent treatment trajectories consistent with findings from the two bodies of literature independently. Previous studies have similarly identified distinct depression symptom trajectories among patients with depression, although they differ slightly from our results. One study measured PHQ9 for one year [11] and identified five distinct trajectories, only one of which was decreasing. These results differ from our three-group model, which may be partially explained by their study not providing an intervention to all participants. Another study measured PHQ9 during psychological treatment for anxiety and depression [16] and identified four trajectories, three of which decreased to a mild PHQ9 threshold. They identified 28% whose depression scores remained high at a “severe” depression level, which may be partially explained by their participants’ increased clinical complexity given their co-occurring anxiety. Moreover, the differences between our findings and previous studies may be explained by our modeling the two outcomes jointly, which identifies groups based on both depression symptoms and weight loss.

Obesity trajectories from previous studies reveal similar trends to our results. Zheng et al. identified three groups – “good responders,” “fair responders,” and “poor responders” – when undergoing a behavioral treatment for weight loss [15], similar to our three-group findings. Their analysis revealed a larger portion in the “fair responders” group (n = 176, 52%) than we identified in our “Substantial/Moderate” group (n = 58, 29%), although we similarly found a small minority of participants in their “good responders” group (n = 49, 14%) and our “Substantial/Substantial” group (n = 35, 17%). Similar findings were also found in a study using growth mixture modelling, with largest 12-month BMI loss in the oldest group, just as our results revealed [8]. While our obesity symptom trajectories resemble results from the literature, the most significant contribution of this study is modeling the two outcomes simultaneously. Analyzing simultaneous trajectories of both depression symptom and weight loss outcomes can elucidate patterns of response to the integrated behavioral treatment among a heterogenous sample, thereby informing future prognostic differentiation and treatment optimization.

Our findings also highlight the importance of conducting precision clinical trials and developing strategies for tailoring and adapting interventions [39,40]. Such an approach may involve consistent short-term measurement of outcomes, focused treatment enrichment among participants with expected low response (e.g., the younger participants or those with complex clinical conditions), and tailoring treatments (e.g., augmentation of an intervention with additional support). To this end, implementation of sequential multiple assignment randomized trials (SMART) or other adaptive trial designs would be valuable for improving outcomes in two domains among heterogeneous samples, as identified herein. While these designs are increasingly being used in interventions for either weight [41] or depression [42] independently, their utility for studies aiming to tackle both outcomes simultaneously may be particularly robust as adaptive treatment decisions could be made using information from both domains.

### Limitations

There are several limitations to this analysis. First, the RAINBOW clinical trial was conducted in a single health system in Northern California; participants had high income and were mostly White and female, limiting generalizability to other geographic regions or demographic groups. In addition, we were limited to the information that the trial collected, so additional unmeasured confounders may exist. RAINBOW data collection concluded in 2017 (almost 8 years after the secondary analysis done in this study), so the analysis should be replicated in more recent clinical trials investigating both depression and obesity. PHQ9 score was modeled as a normally distributed, continuous variable, despite being a discrete integer and slightly skewed. We recognize that this may introduce some bias, particularly in the lowest range of the scale where the assumption of normality deviates most.

Finally, this study relied partially on self-report data which results in high missingness. GBMTM is robust to missingness; however, missing almost 50% of PHQ9 scores and almost 80% of weight measurements near the end of the trial may decrease the confidence in our group assignments. While this is an important limitation, the GBMTM probabilities for the three-group model are still relatively high for all groups (ranging from 72.3% to 92.7%), indicating that most individuals with missing data were still able to be assigned to trajectories with high confidence. The group with highest missingness was the group with the smallest weight changes that was used as the reference group for analyses examining 12-month outcomes. It is possible that the missingness in this group’s 12-month outcomes may have skewed the study’s findings in the direction of the null if individuals with missingness tended to have worse outcomes, which would make the true effect estimates larger than those observed herein. Because of this missingness and the wide confidence intervals on the odds ratios, results should be interpreted primarily for their directionality, and potential magnitude of increase in odds of clinically significant changes in weight or depression should be interpreted with caution.

## Conclusion

Three latent groups of participants exhibiting distinct simultaneous trajectories of depression symptom scores and weight loss during treatment were identified. Older participants were most likely to belong to groups with depression symptom reduction and weight loss. Future adaptive trials or SMART designs should be considered, in an attempt to improve treatment outcomes for participants with lower anticipated depression symptom and/or weight reductions.

## Supporting information

S1 Fig Comparing number of groups for multi-model. AIC Plot for Multi-models with one to five groups.
(PNG)

S1 Table AIC and BIC for models with 1–5 classes. AIC and BIC values for Multi-models with one to five groups.
(DOCX)

S2 Table Mean probabilities and odds of correct classification (OCC) of each group in 2, 3, and 4-group model. Measures of model confidence in class assignment for Multi-models with two to four groups.
(DOCX)

S2 Fig PHQ9 individual trajectories and average PHQ9 trajectories. Spaghetti plots of individual PHQ9 trajectories overlaid with average 3-group multimodel trajectories.
(PNG)

S3 Fig Weight individual trajectories and average weight trajectories. Spaghetti plots of individual weight trajectories overlaid with average 3-group multimodel trajectories.
(PNG)

S3 Table Demographics in sensitivity analysis and overall cohort. Summary statistics of demographics and comorbidities across 3 originally identified groups and after filtering to class assignments with >0.5 and >0.7 predicted probability.
(DOCX)

S4 Table Regression results for individuals with at least p = 0.5 class assignment probability. Results from linear and logistic regressions on sensitivity analysis with >0.5 predicted probability.
(DOCX)

S5 Table Regression results for individuals with at least p = 0.7 class assignment probability. Results from linear and logistic regressions on sensitivity analysis with >0.7 predicted probability.
(DOCX)

S4 Fig Sensitivity analysis with threshold = 0.5. Multi-model trajectories with 3-group model and class assignment probability threshold of 0.5.
(PNG)

S5 Fig Sensitivity analysis with threshold = 0.7. Multi-model trajectories with 3-group model and class assignment probability threshold of 0.7.
(PNG)

S6 Fig Missingness over time. Missingness of both repeated-measure variables (PHQ9 and weight) over the study period.
(PNG)

S6 Table Count of observations for each variable at each time point. Assessing missingness of repeated measures (PHQ9, self-measured weight) and primary outcomes (SCL20, lab-measured weight) across the 54 weeks of the study.
(DOCX)

S7 Table Demographics stratified by missingness of outcomes: SCL20 and Weight at 12 months. Group demographics stratified by whether the participant is missing each primary outcome (SCL20, lab-measured weight) at 12 months.
(DOCX)
