# Supplementary figures and images for: Depression and weight loss trajectories during an integrated behavioral intervention: Within-treatment analysis of the RAINBOW trial
Source: PLoS One. 2025 Dec 19;20(12):e0328715. doi: 10.1371/journal.pone.0328715 (PMC12716787; doi:10.1371/journal.pone.0328715)

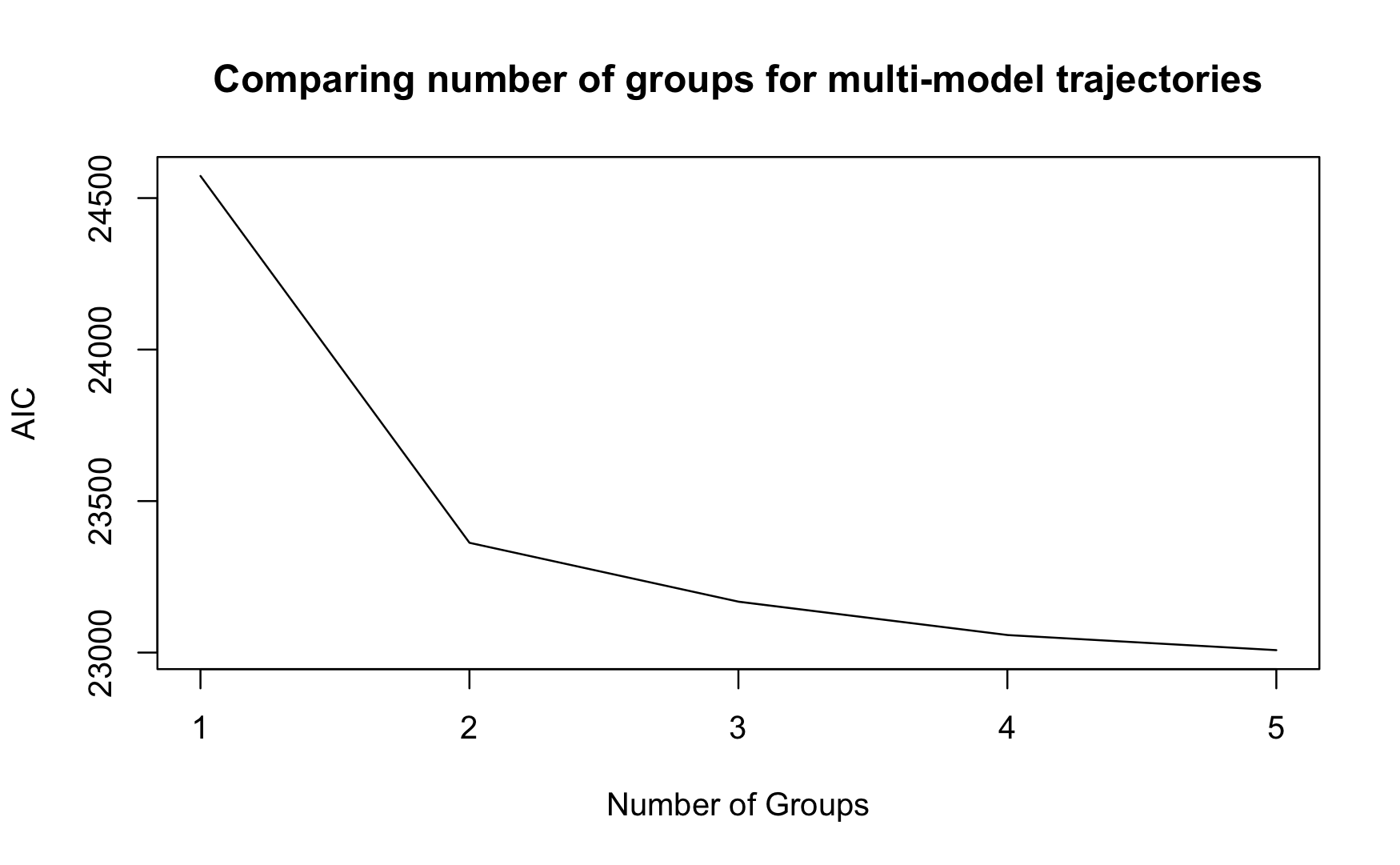

Supplement: S1 Fig — AIC Plot for Multi-models with one to five groups. (PNG) [file pone.0328715.s001.png]

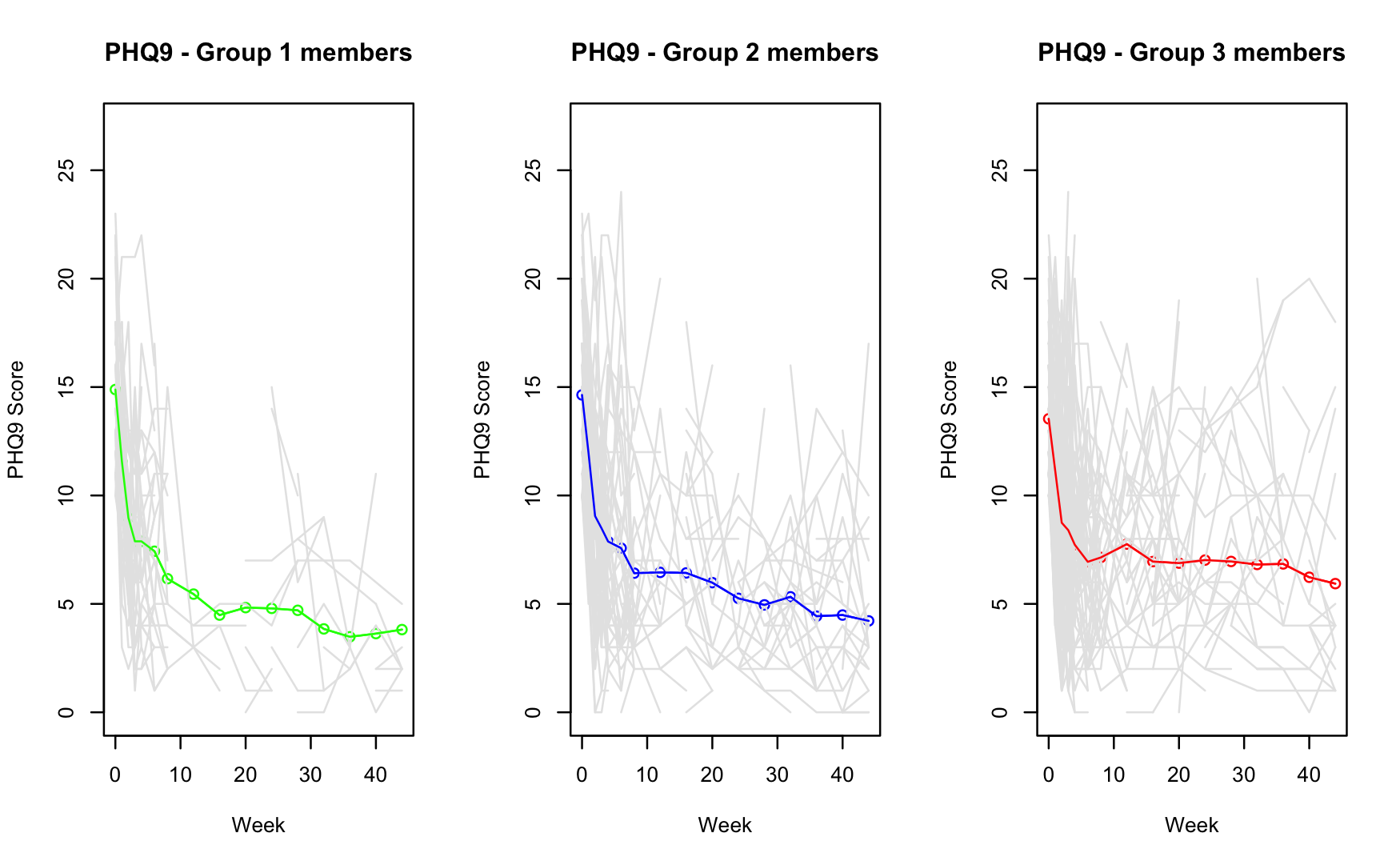

Supplement: S2 Fig — Spaghetti plots of individual PHQ9 trajectories overlaid with average 3-group multimodel trajectories. (PNG) [file pone.0328715.s004.png]

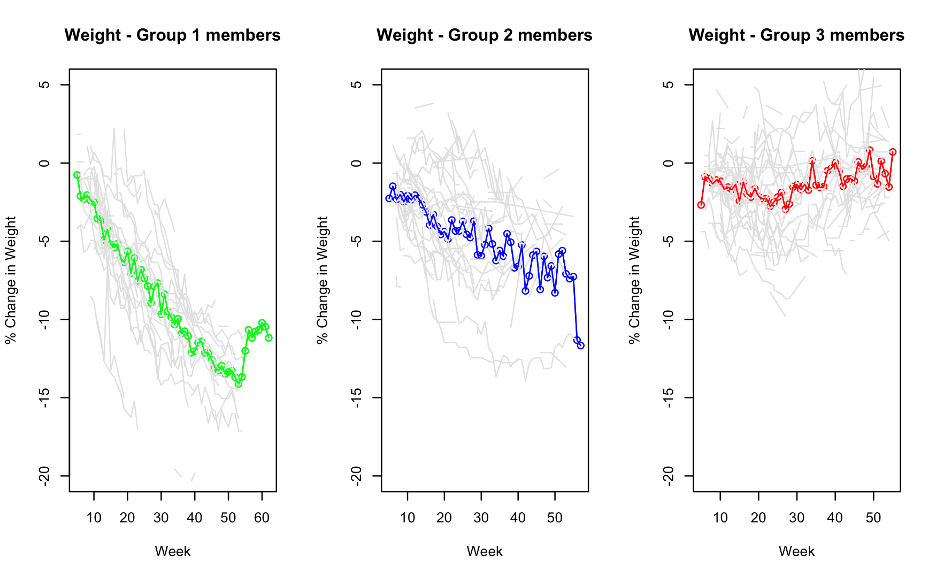

Supplement: S3 Fig — Spaghetti plots of individual weight trajectories overlaid with average 3-group multimodel trajectories. (PNG) [file pone.0328715.s005.png]

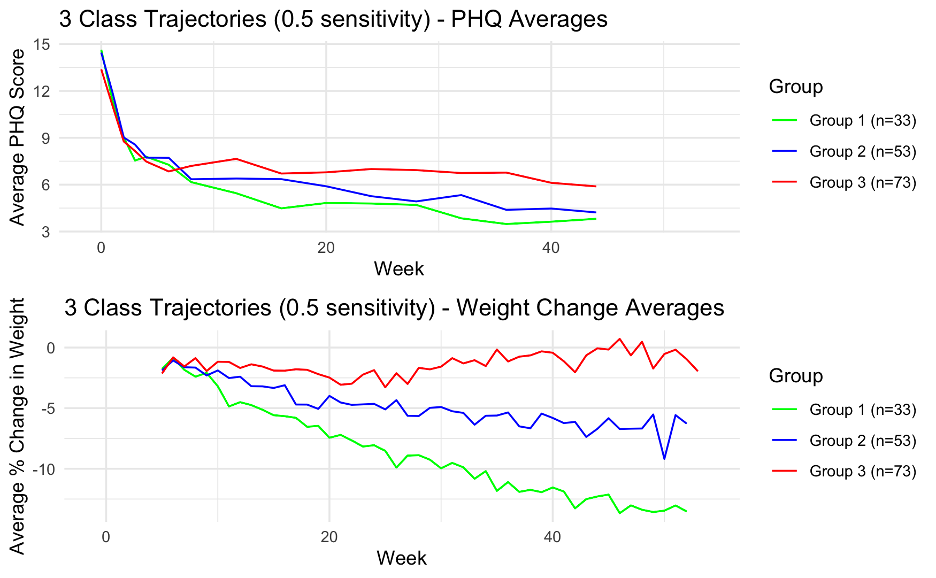

Supplement: S4 Fig — Multi-model trajectories with 3-group model and class assignment probability threshold of 0.5. (PNG) [file pone.0328715.s009.png]

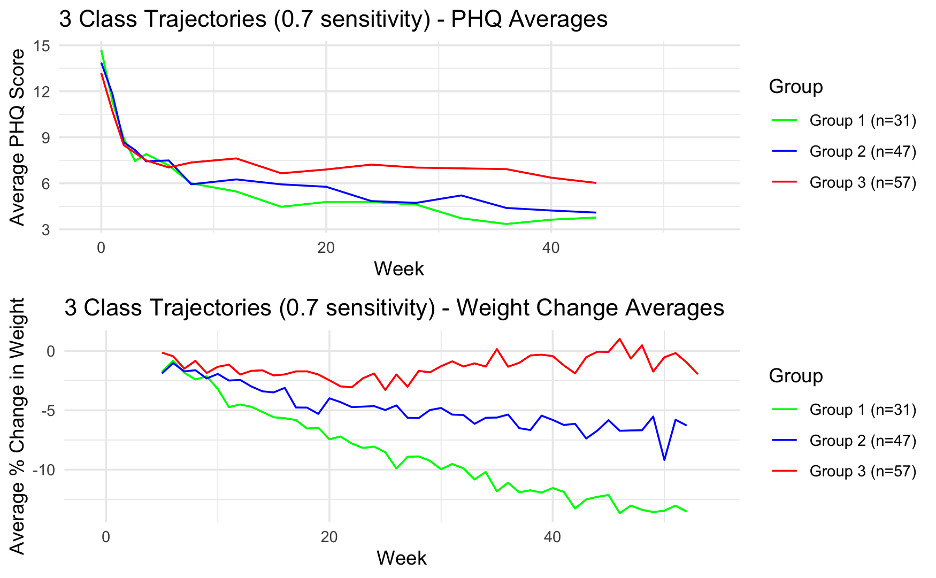

Supplement: S5 Fig — Multi-model trajectories with 3-group model and class assignment probability threshold of 0.7. (PNG) [file pone.0328715.s010.png]

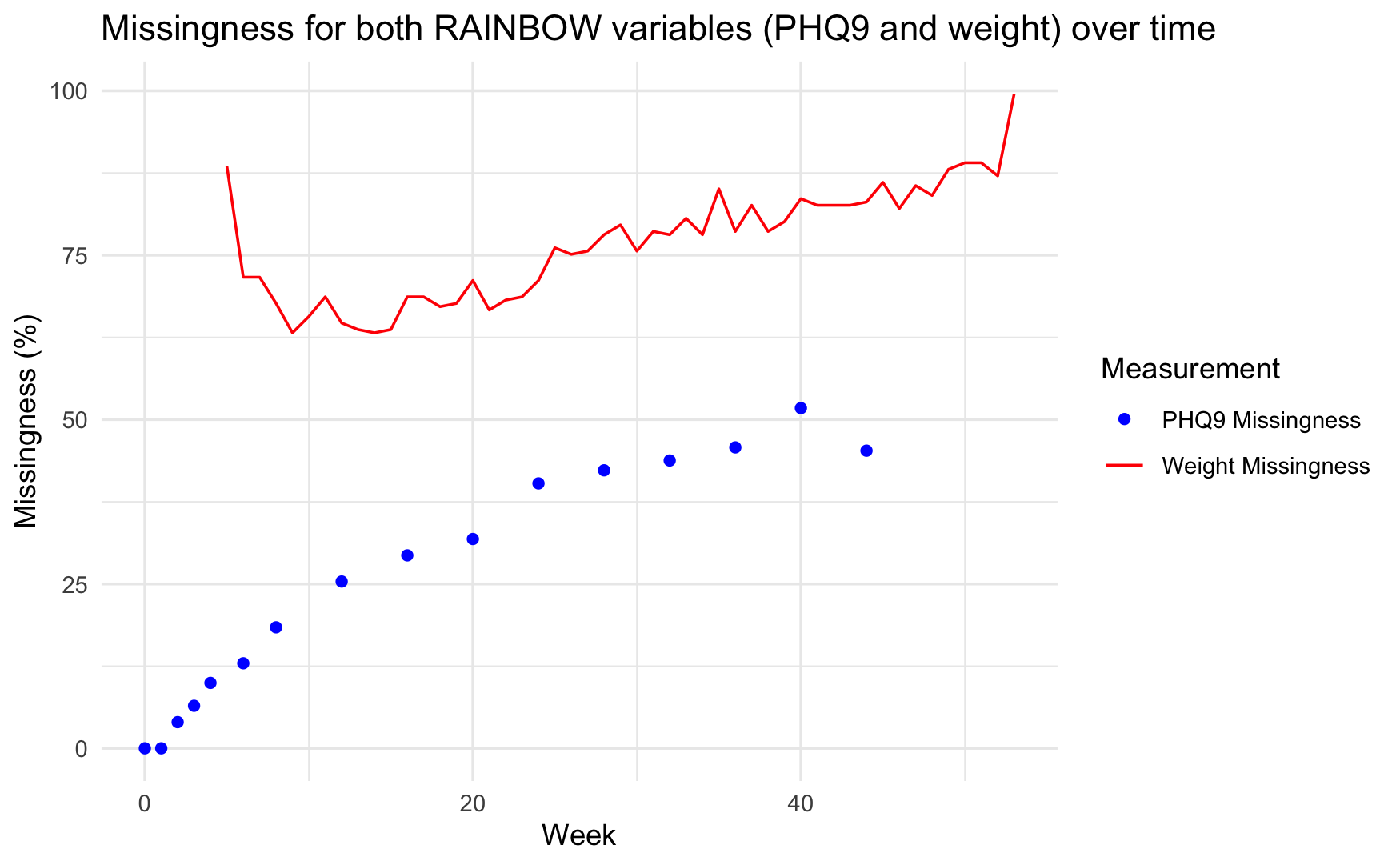

Supplement: S6 Fig — Missingness of both repeated-measure variables (PHQ9 and weight) over the study period. (PNG) [file pone.0328715.s011.png]
