# Supplementary material for: Depression and weight loss trajectories during an integrated behavioral intervention: Within-treatment analysis of the RAINBOW trial
Source: PLoS One. 2025 Dec 19;20(12):e0328715. doi: 10.1371/journal.pone.0328715 (PMC12716787; doi:10.1371/journal.pone.0328715)
Supplement: S1 Table — AIC and BIC values for Multi-models with one to five groups. (DOCX) [file pone.0328715.s002.docx]

| S1 Table: AIC and BIC for models with 1 through 5 classes | | | | | | | | |
| --- | --- | --- | --- | --- | --- | --- | --- | --- |
| # Classes | AIC | BIC | entropy | % class1 | % class2 | % class3 | % class4 | % class5 |
| 1 | 24572.93 | 24596.05 | 1.00 | 100.00 | NA | NA | NA | NA |
| 2 | 23362.55 | 23395.58 | 0.69 | 24.88 | 75.12 | NA | NA | NA |
| 3 | 23168.13 | 23211.07 | 0.62 | 17.41 | 28.86 | 53.73 | NA | NA |
| 4 | 23058.02 | 23110.87 | 0.63 | 7.46 | 31.34 | 9.45 | 51.74 | NA |
| 5 | 23008.03 | 23070.8 | 0.66 | 6.97 | 53.23 | 26.37 | 9.95 | 3.48 |
