# Supplementary material for: Depression and weight loss trajectories during an integrated behavioral intervention: Within-treatment analysis of the RAINBOW trial
Source: PLoS One. 2025 Dec 19;20(12):e0328715. doi: 10.1371/journal.pone.0328715 (PMC12716787; doi:10.1371/journal.pone.0328715)
Supplement: S2 Table — Measures of model confidence in class assignment for Multi-models with two to four groups. (DOCX) [file pone.0328715.s003.docx]

| S2 Table – Mean probabilities and odds of correct classification (OCC) of each group in 2, 3, and 4-group model | | | | |
| --- | --- | --- | --- | --- |
| **Model** | **Group 1**  **Mean Probability (OCC)** | **Group 2**  **Mean Probability (OCC)** | **Group 3**  **Mean Probability (OCC)** | **Group 4**  **Mean Probability (OCC)** |
| 2-group model | 0.92  (35.16) | 0.88  (2.48) |  |  |
| 3-group model | 0.93  (60.48) | 0.85  (13.84) | 0.72  (2.26) |  |
| 4-group model | 0.86  (74.16) | 0.79  (8.24) | 0.93  (119.17) | 0.71  (2.29) |
