# Supplementary material for: Depression and weight loss trajectories during an integrated behavioral intervention: Within-treatment analysis of the RAINBOW trial
Source: PLoS One. 2025 Dec 19;20(12):e0328715. doi: 10.1371/journal.pone.0328715 (PMC12716787; doi:10.1371/journal.pone.0328715)
Supplement: S3 Table — Summary statistics of demographics and comorbidities across 3 originally identified groups and after filtering to class assignments with >0.5 and >0.7 predicted probability. (DOCX) [file pone.0328715.s006.docx]

| **Table S3: Demographics in sensitivity analysis and overall cohort** | | | |
| --- | --- | --- | --- |
|  | **Sensitivity Analysis - 0.5** | **Sensitivity Analysis - 0.7** | **Overall** |
|  | **(N=159)** | **(N=135)** | **(N=201)** |
| **Baseline Weight** | | | |
| Median [Min, Max] | 99.8 [61.1, 182] | 99.9 [61.1, 182] | 99.8 [61.1, 203] |
| **Baseline SCL20** | | | |
| Median [Min, Max] | 1.50 [0.300, 2.65] | 1.40 [0.300, 2.65] | 1.45 [0.300, 2.65] |
| **Sex** | | | |
| Men | 46 (28.9%) | 40 (29.6%) | 59 (29.4%) |
| Women | 113 (71.1%) | 95 (70.4%) | 142 (70.6%) |
| **Race** | | | |
| Asian | 14 (8.8%) | 12 (8.9%) | 18 (9.0%) |
| Black | 2 (1.3%) | 2 (1.5%) | 3 (1.5%) |
| Hispanic | 19 (11.9%) | 16 (11.9%) | 26 (12.9%) |
| Non-Hispanic White | 117 (73.6%) | 100 (74.1%) | 146 (72.6%) |
| Other | 7 (4.4%) | 5 (3.7%) | 8 (4.0%) |
| **Age** | | | |
| Median [Min, Max] | 52.7 [21.8, 76.0] | 53.7 [22.8, 76.0] | 51.8 [20.0, 76.0] |
| **Education** | | | |
| < High school or GED | 8 (5.0%) | 6 (4.4%) | 10 (5.0%) |
| Some college | 39 (24.5%) | 32 (23.7%) | 51 (25.4%) |
| Undergraduate degree | 67 (42.1%) | 57 (42.2%) | 77 (38.3%) |
| Graduate work or degree | 45 (28.3%) | 40 (29.6%) | 63 (31.3%) |
| **Income** | | | |
| $0 to $9,999 | 1 (0.6%) | 1 (0.7%) | 1 (0.5%) |
| $10,000 to $19,999 | 3 (1.9%) | 3 (2.2%) | 5 (2.5%) |
| $20,000 to $34,999 | 4 (2.5%) | 4 (3.0%) | 4 (2.0%) |
| $35,000 to $54,999 | 12 (7.5%) | 11 (8.1%) | 14 (7.0%) |
| $55,000 to $74,999 | 17 (10.7%) | 13 (9.6%) | 22 (10.9%) |
| $75,000 to $99,999 | 15 (9.4%) | 11 (8.1%) | 20 (10.0%) |
| $100,000 to $124,999 | 16 (10.1%) | 12 (8.9%) | 20 (10.0%) |
| $125,000 to $149,999 | 14 (8.8%) | 12 (8.9%) | 14 (7.0%) |
| $150,000+ | 56 (35.2%) | 49 (36.3%) | 74 (36.8%) |
| Missing | 21 | 19 | 227 |
| **Marital Status** | | | |
| Married/living with another person | 96 (60.4%) | 83 (61.5%) | 120 (59.7%) |
| Single | 62 (39.0%) | 51 (37.8%) | 80 (39.8%) |
| Missing | 1 | 1 | 1 |
| **SBP** | | | |
| Median [Min, Max] | 120 [90.0, 160] | 120 [90.0, 160] | 120 [90.0, 160] |
| **DBP** | | | |
| Median [Min, Max] | 79.3 [58.7, 104] | 78.7 [61.3, 104] | 78.7 [58.7, 104] |
| **Sheehan Disability** | | | |
| Median [Min, Max] | 11.0 [0, 30.0] | 10.0 [0, 30.0] | 11.0 [0, 30.0] |
| Missing | 2 | 2 | 3 |
| **Calories** | | | |
| Median [Min, Max] | 1700 [618, 5150] | 1700 [618, 5150] | 1690 [273, 5150] |
| Missing | 1 | 1 | 2 |
| **Physical Activity** | | | |
| Median [Min, Max] | 32.8 [28.8, 48.0] | 32.9 [30.6, 48.0] | 32.8 [28.8, 48.0] |
| **Obesity Problems Score** | | | |
| Median [Min, Max] | 70.8 [4.17, 100] | 70.8 [4.17, 100] | 70.8 [0, 100] |
| **Binge Eating Disorder** | | | |
| Yes | 62 (39.0%) | 55 (40.7%) | 76 (37.8%) |
| **Panic Disorder** | | | |
| None | 131 (82.4%) | 113 (83.7%) | 165 (82.1%) |
| Lifetime | 14 (8.8%) | 10 (7.4%) | 20 (10.0%) |
| Limited symptom attacks lifetime | 0 (0%) | 0 (0%) | 1 (0.5%) |
| Current | 11 (6.9%) | 11 (8.1%) | 11 (5.5%) |
| Missing | 3 | 1 | 4 |
| **Any antidepressant medications** | | | |
| Yes | 20 (12.6%) | 18 (13.3%) | 24 (11.9%) |
| **PTSD Score** | | | |
| Median [Min, Max] | 36.0 [19.0, 78.0] | 36.0 [19.0, 75.0] | 36.0 [17.0, 78.0] |
| Missing | 1 | 1 | 2 |
| **GAD7 Score** | | | |
| Median [Min, Max] | 7.00 [0, 21.0] | 7.00 [0, 21.0] | 7.50 [0, 21.0] |
| Missing | 0 | 0 | 1 |
| **Alcohol Use** | | | |
| Yes | 90 (56.6%) | 75 (55.6%) | 108 (53.7%) |
| No | 50 (31.4%) | 42 (31.1%) | 70 (34.8%) |
| Missing | 19 | 18 | 23 |
| **Tobacco Use** | | | |
| Yes | 6 (3.8%) | 4 (3.0%) | 8 (4.0%) |
| Quit | 41 (25.8%) | 33 (24.4%) | 49 (24.4%) |
| Never | 107 (67.3%) | 93 (68.9%) | 139 (69.2%) |
| Missing | 5 | 5 | 5 |
