# Supplementary material for: Depression and weight loss trajectories during an integrated behavioral intervention: Within-treatment analysis of the RAINBOW trial
Source: PLoS One. 2025 Dec 19;20(12):e0328715. doi: 10.1371/journal.pone.0328715 (PMC12716787; doi:10.1371/journal.pone.0328715)
Supplement: S4 Table — Results from linear and logistic regressions on sensitivity analysis with >0.5 predicted probability. (DOCX) [file pone.0328715.s007.docx]

| S4 Table – regression results for individuals with at least p=0.5 class assignment probability | | | | | | |
| --- | --- | --- | --- | --- | --- | --- |
|  | **Linear Regression Results** | | | **Logistic Regression Results** | | |
| **SCL20 (observations** **= 143)** | | | | | | |
| *Predictors* | *Unadjusted SCL change*  *(SD)* | *Estimates* | *p* | *Proportion achieving clinically significant SCL* | *Odds Ratios* | *p* |
| Baseline SCL20 | - | -0.58 (-0.79, -0.37) | **<0.001** | - | 1.48 (0.72, 3.09) | 0.292 |
| Age | - | -0.00 (-0.01, 0.00) | 0.307 | - | 1.02 (0.99, 1.06) | 0.173 |
| Moderate / Minimal (n=64) | -0.14  (0.81) | Ref | Ref | 0.21 | Ref | Ref |
| Substantial / Moderate (n=49) | -0.51  (0.60) | -0.31 (-0.55, -0.06) | **0.014** | 0.30 | 1.46 (0.63, 3.43) | 0.378 |
| Substantial / Substantial (n=30) | -0.57 (0.64) | -0.46 (-0.75, -0.17) | **0.002** | 0.45 | 3.06 (1.20, 7.97) | **0.020** |
| **Weight (observations = 148)** | | | | | | |
| *Predictors* | *Unadjusted Weight change*  *(SD)* | *Estimates* | *P* | *Proportion achieving clinically significant Weight* | *Odds Ratios* | *p* |
| Baseline Weight (kg) | - | -0.03 (-0.06, 0.01) | 0.098 | - | 1.00 (0.97, 1.02) | 0.935 |
| Age | - | 0.02 (-0.04, 0.08) | 0.470 | - | 1.01 (0.96, 1.05) | 0.747 |
| Moderate / Minimal (n=69) | 1.35 (4.55) | Ref | Ref | 0.03 | Ref | Ref |
| Substantial / Moderate (n=49) | -3.32  (3.70) | -4.70 (-6.29, -3.11) | **<0.001** | 0.28 | 14.56 (3.80, 96.09) | **0.001** |
| Substantial / Substantial (n=30) | -9.74  (4.65) | -11.44 (-13.34, -9.55) | **<0.001** | 0.82 | 288.20 (55.94, 2535.75) | **<0.001** |
