# Supplementary material for: Depression and weight loss trajectories during an integrated behavioral intervention: Within-treatment analysis of the RAINBOW trial
Source: PLoS One. 2025 Dec 19;20(12):e0328715. doi: 10.1371/journal.pone.0328715 (PMC12716787; doi:10.1371/journal.pone.0328715)
Supplement: S5 Table — Results from linear and logistic regressions on sensitivity analysis with >0.7 predicted probability. (DOCX) [file pone.0328715.s008.docx]

| S5 Table – regression results for individuals with at least p=0.7 class assignment probability | | | | | | |
| --- | --- | --- | --- | --- | --- | --- |
|  | **Linear Regression Results** | | | **Logistic Regression Results** | | |
| **SCL20 (observations** **=124)** | | | | | | |
| *Predictors* | *Unadjusted SCL change*  *(SD)* | *Estimates* | *p* | *Proportion achieving clinically significant SCL* | *Odds Ratios* | *p* |
| Baseline SCL20 | - | -0.59  (-0.8, -0.38) | **<0.001** | - | 1.42  (0.68, 3.03) | 0.354 |
| Age | - | -0.01  (-0.01, 0) | 0.248 | - | 1.026  (0.99, 1.06) | 0.128 |
| Moderate / Minimal (n=53) | -0.20 (0.74) | Ref | Ref | 0.25 | Ref |  |
| Substantial / Moderate (n=43) | -0.52 (0.59) | -0.29  (-0.54, -0.05) | 0.021 | 0.32 | 1.403  (0.57, 3.44) | 0.456 |
| Substantial / Substantial (n=28) | -0.61 (0.65) | -0.42  (-0.7, -0.13) | 0.00417 | 0.48 | 2.944  (1.11, 8.01) | 0.031 |
| **Weight (observations =126)** | | | | | | |
| *Predictors* | *Unadjusted Weight change*  *(SD)* | *Estimates* | *p* | *Proportion achieving clinically significant Weight* | *Odds Ratios* | *p* |
| Baseline Weight (kg) | - | -0.03  (-0.06, 0) | 0.062 | - | 0.997  (0.97, 1.03) | 0.828 |
| Age | - | 0.01  (-0.05, 0.06) | 0.818 | - | 0.996  (0.94, 1.05) | 0.876 |
| Moderate / Minimal (n=54) | 1.36 (3.43) | Ref | Ref | 0.02 | Ref | Ref |
| Substantial / Moderate (n=44) | -3.54 (3.77) | -4.87  (-6.37, -3.36) | **<0.001** | 0.32 | 27.923  (5.2, 520.51) | 0.002 |
| Substantial / Substantial (n=28) | -10.30 (4.29) | -11.96  (-13.72, -10.2) | **<0.001** | 0.87 | 1432.5  (138.5, 53833.41) | **<0.001** |
