# Supplementary material for: Depression and weight loss trajectories during an integrated behavioral intervention: Within-treatment analysis of the RAINBOW trial
Source: PLoS One. 2025 Dec 19;20(12):e0328715. doi: 10.1371/journal.pone.0328715 (PMC12716787; doi:10.1371/journal.pone.0328715)
Supplement: S6 Table — Assessing missingness of repeated measures (PHQ9, self-measured weight) and primary outcomes (SCL20, lab-measured weight) across the 54 weeks of the study. (DOCX) [file pone.0328715.s012.docx]

| **Table S6: Count of observations for each variable at each time point**  **n (%)** | | | | |
| --- | --- | --- | --- | --- |
|  | **Repeated Measures** | | **Primary Outcomes** | |
| **Week**   **(joined to PHQ9 sessions)** | **PHQ9** | **Self-measured Weight** | **SCL20** | **Lab-measured weight** |
| Baseline | NA | NA | 201 (100%) | 201 (100%) |
| 0 | 201 (100%) | 0 (0%) | NA | NA |
| 1 | 201 (100%) | 0 (0%) | NA | NA |
| 2 | 193 (96.02%) | 0 (0%) | NA | NA |
| 3 | 188 (93.53%) | 0 (0%) | NA | NA |
| 4 | 181 (90.05%) | 0 (0%) | NA | NA |
| 5 | 0 (0%) | 23 (11.44%) | NA | NA |
| 6 | 175 (87.06%) | 57 (28.36%) | NA | NA |
| 7 | 0 (0%) | 57 (28.36%) | NA | NA |
| 8 | 164 (81.59%) | 65 (32.34%) | NA | NA |
| 9 | 0 (0%) | 74 (36.82%) | NA | NA |
| 10 | 0 (0%) | 69 (34.33%) | NA | NA |
| 11 | 0 (0%) | 63 (31.34%) | NA | NA |
| 12 | 150 (74.63%) | 71 (35.32%) | NA | NA |
| 13 | 0 (0%) | 73 (36.32%) | NA | NA |
| 14 | 0 (0%) | 74 (36.82%) | NA | NA |
| 15 | 0 (0%) | 73 (36.32%) | NA | NA |
| 16 | 142 (70.65%) | 63 (31.34%) | NA | NA |
| 17 | 0 (0%) | 63 (31.34%) | NA | NA |
| 18 | 0 (0%) | 66 (32.84%) | NA | NA |
| 19 | 0 (0%) | 65 (32.34%) | NA | NA |
| 20 | 137 (68.16%) | 58 (28.86%) | NA | NA |
| 21 | 0 (0%) | 67 (33.33%) | NA | NA |
| 22 | 0 (0%) | 64 (31.84%) | NA | NA |
| 23 | 0 (0%) | 63 (31.34%) | NA | NA |
| 24 | 120 (59.7%) | 58 (28.86%) | NA | NA |
| 25 | 0 (0%) | 48 (23.88%) | NA | NA |
| 26 | 0 (0%) | 50 (24.88%) | NA | NA |
| 27 | 0 (0%) | 49 (24.38%) | NA | NA |
| 28 | 116 (57.71%) | 44 (21.89%) | NA | NA |
| 29 | 0 (0%) | 41 (20.4%) | NA | NA |
| 30 | 0 (0%) | 49 (24.38%) | NA | NA |
| 31 | 0 (0%) | 43 (21.39%) | NA | NA |
| 32 | 113 (56.22%) | 44 (21.89%) | NA | NA |
| 33 | 0 (0%) | 39 (19.4%) | NA | NA |
| 34 | 0 (0%) | 44 (21.89%) | NA | NA |
| 35 | 0 (0%) | 30 (14.93%) | NA | NA |
| 36 | 109 (54.23%) | 43 (21.39%) | NA | NA |
| 37 | 0 (0%) | 35 (17.41%) | NA | NA |
| 38 | 0 (0%) | 43 (21.39%) | NA | NA |
| 39 | 0 (0%) | 40 (19.9%) | NA | NA |
| 40 | 97 (48.26%) | 33 (16.42%) | NA | NA |
| 41 | 0 (0%) | 35 (17.41%) | NA | NA |
| 42 | 0 (0%) | 35 (17.41%) | NA | NA |
| 43 | 0 (0%) | 35 (17.41%) | NA | NA |
| 44 | 110 (54.73%) | 34 (16.92%) | NA | NA |
| 45 | 0 (0%) | 28 (13.93%) | NA | NA |
| 46 | 0 (0%) | 36 (17.91%) | NA | NA |
| 47 | 0 (0%) | 29 (14.43%) | NA | NA |
| 48 | 0 (0%) | 32 (15.92%) | NA | NA |
| 49 | 0 (0%) | 24 (11.94%) | NA | NA |
| 50 | 0 (0%) | 22 (10.95%) | NA | NA |
| 51 | 0 (0%) | 22 (10.95%) | NA | NA |
| 52 | 0 (0%) | 26 (12.94%) | NA | NA |
| 53 | 0 (0%) | 1 (0.5%) | NA | NA |
| Final 12-month follow up | NA | NA | 169 (84.08%) | 180 (89.55%) |
