# Supplementary material for: Depression and weight loss trajectories during an integrated behavioral intervention: Within-treatment analysis of the RAINBOW trial
Source: PLoS One. 2025 Dec 19;20(12):e0328715. doi: 10.1371/journal.pone.0328715 (PMC12716787; doi:10.1371/journal.pone.0328715)
Supplement: S7 Table — Group demographics stratified by whether the participant is missing each primary outcome (SCL20, lab-measured weight) at 12 months. (DOCX) [file pone.0328715.s013.docx]

| **Table S7: Demographics stratified by missingness of outcomes: SCL20 and Weight at 12 months** | | | | | |
| --- | --- | --- | --- | --- | --- |
|  | **Missing 12-month SCL observation** | **Has 12-month SCL observation** | **Missing 12-month weight observation** | **Has 12-month Weight observation** | **Overall** |
|  | **(N=32)** | **(N=169)** | **(N=21)** | **(N=180)** | **(N=201)** |
| **Baseline Weight** | | | | | |
| Median [Min, Max] | 104 [82.6, 163] | 98.2 [61.1, 203] | 99.5 [82.8, 163] | 99.9 [61.1, 203] | 99.8 [61.1, 203] |
| **Baseline SCL20** | | | | | |
| Median [Min, Max] | 1.73 [0.350, 2.60] | 1.40 [0.300, 2.65] | 1.65 [0.600, 2.60] | 1.43 [0.300, 2.65] | 1.45 [0.300, 2.65] |
| **Trajectory assigned** | | | | | |
| 1 (Substantial / Substantial) | 4 (12.5%) | 31 (18.3%) | 4 (19.0%) | 31 (17.2%) | 35 (17.4%) |
| 2 (Substantial / Moderate) | 5 (15.6%) | 53 (31.4%) | 4 (19.0%) | 54 (30.0%) | 58 (28.9%) |
| 3 (Moderate / Minimal) | 23 (71.9%) | 85 (50.3%) | 13 (61.9%) | 95 (52.8%) | 108 (53.7%) |
| **Sex** | | | | | |
| Women | 20 (62.5%) | 122 (72.2%) | 13 (61.9%) | 129 (71.7%) | 142 (70.6%) |
| Men | 12 (37.5%) | 47 (27.8%) | 8 (38.1%) | 51 (28.3%) | 59 (29.4%) |
| **Race** | | | | | |
| Asian | 2 (6.3%) | 16 (9.5%) | 2 (9.5%) | 16 (8.9%) | 18 (9.0%) |
| Hispanic | 3 (9.4%) | 23 (13.6%) | 2 (9.5%) | 24 (13.3%) | 26 (12.9%) |
| Non-Hispanic White | 24 (75.0%) | 122 (72.2%) | 15 (71.4%) | 131 (72.8%) | 146 (72.6%) |
| Other | 3 (9.4%) | 5 (3.0%) | 2 (9.5%) | 6 (3.3%) | 8 (4.0%) |
| Black | 0 (0%) | 3 (1.8%) | 0 (0%) | 3 (1.7%) | 3 (1.5%) |
| **Age** | | | | | |
| Median [Min, Max] | 51.5 [20.0, 70.9] | 51.8 [22.8, 76.0] | 53.0 [24.6, 70.9] | 51.8 [20.0, 76.0] | 51.8 [20.0, 76.0] |
| **Education** | | | | | |
| < High school or GED | 1 (3.1%) | 9 (5.3%) | 1 (4.8%) | 9 (5.0%) | 10 (5.0%) |
| Some college | 11 (34.4%) | 40 (23.7%) | 5 (23.8%) | 46 (25.6%) | 51 (25.4%) |
| Undergraduate degree | 13 (40.6%) | 64 (37.9%) | 9 (42.9%) | 68 (37.8%) | 77 (38.3%) |
| Graduate work or degree | 7 (21.9%) | 56 (33.1%) | 6 (28.6%) | 57 (31.7%) | 63 (31.3%) |
| **Income** | | | | | |
| $0 to $9,999 | 0 (0%) | 1 (0.6%) | 0 (0%) | 1 (0.6%) | 1 (0.5%) |
| $10,000 to $19,999 | 0 (0%) | 5 (3.0%) | 0 (0%) | 5 (2.8%) | 5 (2.5%) |
| $20,000 to $34,999 | 0 (0%) | 4 (2.4%) | 0 (0%) | 4 (2.2%) | 4 (2.0%) |
| $35,000 to $54,999 | 3 (9.4%) | 11 (6.5%) | 1 (4.8%) | 13 (7.2%) | 14 (7.0%) |
| $55,000 to $74,999 | 2 (6.3%) | 20 (11.8%) | 0 (0%) | 22 (12.2%) | 22 (10.9%) |
| $75,000 to $99,999 | 2 (6.3%) | 18 (10.7%) | 0 (0%) | 20 (11.1%) | 20 (10.0%) |
| $100,000 to $124,999 | 4 (12.5%) | 16 (9.5%) | 3 (14.3%) | 17 (9.4%) | 20 (10.0%) |
| $125,000 to $149,999 | 1 (3.1%) | 13 (7.7%) | 1 (4.8%) | 13 (7.2%) | 14 (7.0%) |
| $150,000+ | 15 (46.9%) | 59 (34.9%) | 11 (52.4%) | 63 (35.0%) | 74 (36.8%) |
| Decline to state | 5 (15.6%) | 21 (12.4%) | 5 (23.8%) | 21 (11.7%) | 26 (12.9%) |
| Missing | 0 | 1 | 0 | 1 | 1 |
| **Marital Status** | | | | | |
| Married/living with another person | 20 (62.5%) | 100 (59.2%) | 13 (61.9%) | 107 (59.4%) | 120 (59.7%) |
| Single | 12 (37.5%) | 68 (40.2%) | 8 (38.1%) | 72 (40.0%) | 80 (39.8%) |
| Missing | 0 | 1 | 0 | 1 | 1 |
| **SBP** | | | | | |
| Median [Min, Max] | 115 [101, 160] | 120 [90.0, 148] | 118 [101, 160] | 120 [90.0, 148] | 120 [90.0, 160] |
| **DBP** | | | | | |
| Median [Min, Max] | 75.3 [63.3, 93.3] | 80.0 [58.7, 104] | 77.3 [64.7, 94.7] | 78.7 [58.7, 104] | 78.7 [58.7, 104] |
| **Sheehan Disability** | | | | | |
| Median [Min, Max] | 11.0 [0, 25.0] | 10.0 [0, 30.0] | 11.0 [0, 25.0] | 11.0 [0, 30.0] | 11.0 [0, 30.0] |
| Missing | 1 | 2 | 0 | 3 | 3 |
| **Calories** | | | | | |
| Median [Min, Max] | 1720 [635, 4760] | 1690 [273, 5150] | 1740 [635, 3140] | 1690 [273, 5150] | 1690 [273, 5150] |
| Missing | 1 | 1 | 1 | 1 | 2 |
| **Physical Activity** | | | | | |
| Median [Min, Max] | 32.7 [30.5, 40.3] | 32.9 [28.8, 48.0] | 32.8 [30.6, 36.7] | 32.8 [28.8, 48.0] | 32.8 [28.8, 48.0] |
| **Obesity Problems Score** | | | | | |
| Median [Min, Max] | 66.7 [8.33, 100] | 70.8 [0, 100] | 70.8 [45.8, 100] | 70.8 [0, 100] | 70.8 [0, 100] |
| **Binge Eating Disorder** | | | | | |
| Yes | 14 (43.8%) | 62 (36.7%) | 10 (47.6%) | 66 (36.7%) | 76 (37.8%) |
| **Panic Disorder** | | | | | |
| None | 26 (81.3%) | 139 (82.2%) | 17 (81.0%) | 148 (82.2%) | 165 (82.1%) |
| Lifetime | 4 (12.5%) | 16 (9.5%) | 2 (9.5%) | 18 (10.0%) | 20 (10.0%) |
| Limited symptom attacks lifetime | 1 (3.1%) | 0 (0%) | 1 (4.8%) | 0 (0%) | 1 (0.5%) |
| Current | 1 (3.1%) | 10 (5.9%) | 1 (4.8%) | 10 (5.6%) | 11 (5.5%) |
| Missing | 0 | 4 | 0 | 4 | 4 |
| **Any antidepressant medications** | | | | | |
| Yes | 4 (12.5%) | 20 (11.8%) | 3 (14.3%) | 21 (11.7%) | 24 (11.9%) |
| **PTSD Score** | | | | | |
| Median [Min, Max] | 36.0 [22.0, 73.0] | 36.0 [17.0, 78.0] | 36.0 [25.0, 66.0] | 36.0 [17.0, 78.0] | 36.0 [17.0, 78.0] |
| Missing | 0 | 2 | 0 | 2 | 2 |
| **GAD7 Score** | | | | | |
| Median [Min, Max] | 9.00 [0, 18.0] | 7.00 [0, 21.0] | 9.00 [1.00, 18.0] | 7.00 [0, 21.0] | 7.50 [0, 21.0] |
| Missing | 0 | 1 | 0 | 1 | 1 |
| **Alcohol Use** | | | | | |
| Yes | 18 (56.3%) | 90 (53.3%) | 13 (61.9%) | 95 (52.8%) | 108 (53.7%) |
| No | 11 (34.4%) | 59 (34.9%) | 6 (28.6%) | 64 (35.6%) | 70 (34.8%) |
| Missing | 3 | 20 | 2 | 21 | 23 |
| **Tobacco Use** | | | | | |
| Yes | 3 (9.4%) | 5 (3.0%) | 2 (9.5%) | 6 (3.3%) | 8 (4.0%) |
| Quit | 5 (15.6%) | 44 (26.0%) | 1 (4.8%) | 48 (26.7%) | 49 (24.4%) |
| Never | 24 (75.0%) | 115 (68.0%) | 18 (85.7%) | 121 (67.2%) | 139 (69.2%) |
| Missing | 0 | 5 | 0 | 5 | 5 |
